# Hypoxia and EGF Stimulation Regulate VEGF Expression in Human Glioblastoma Multiforme (GBM) Cells by Differential Regulation of the PI3K/Rho-GTPase and MAPK Pathways

**DOI:** 10.3390/cells8111397

**Published:** 2019-11-06

**Authors:** Samer Nicolas, Sandra Abdellatef, Maria Al Haddad, Isabelle Fakhoury, Mirvat El-Sibai

**Affiliations:** Department of Natural Sciences, Lebanese American University, Beirut 1102 2801, Lebanon; samer.nicolas@lau.edu (S.N.); sandra.abdellatef@lau.edu (S.A.); maria.alhaddad@lau.edu (M.A.H.); isabelle.fakhoury@lau.edu.lb (I.F.)

**Keywords:** glioblastoma, VEGF, hypoxia, EGF, PI3K, MAPK, Rho-GTPases

## Abstract

Glioblastoma multiforme (GBM) is one of the most common and deadly cancers of the central nervous system (CNS). It is characterized by the presence of hypoxic regions, especially in the core, leading to an increase in vascularity. This increased vascularization is driven by the expression of the major angiogenic inducer VEGF and the indirect angiogenic inducer Epidermal growth factor (EGF), which stimulates VEGF expression. In this study, we examine the regulation of VEGF by both hypoxia and the EGF signaling pathway. We also examine the involvement of pathways downstream from EGF signaling, including the mitogen-activated protein kinase/extracellular regulated kinase (MAPK/ERK) pathway and the Phosphatidylinositol-3-kinase/RhoA/C (PI3K/RhoA/C) pathway in this regulation. Our results show that VEGF expression and secretion levels increase following either hypoxia or EGF stimulation, with the two stimuli signaling in parallel. We also observed an increase in ERK and protein kinase B (Akt) phosphorylation, in response to EGF stimulation, with kinetics that correlated with the kinetics of the effect on VEGF. Using pharmacological inhibitors against ERK and PI3K and small interfering RNAs (siRNAs) against RhoA and RhoC, we found that both the ERK and the PI3K/RhoA/C pathways have to cooperate in order to lead to an increase in VEGF expression, downstream from EGF. In response to hypoxia, however, only ERK was involved in the regulation of VEGF. Hypoxia also led to a surprising decrease in the activation of PI3K and RhoA/C. Finally, the decrease in the activation of these Rho-GTPases was found to be mediated through a hypoxia-driven overexpression of the Rho-GTPase GTPase activating protein (GAP), StarD13. Therefore, while under normoxic conditions, EGF stimulates the activation of both the PI3K and the MAPK pathways and the induction of VEGF, in glioblastoma cells, hypoxic conditions lead to the suppression of the PI3K/RhoA/C pathway and an exclusive switch to the MAPK pathway.

## 1. Introduction

Glioblastoma multiforme (GBM) is a malignant brain tumor that originates within the central nervous system (CNS) [1,2]. Despite medical and scientific advancements in cancer therapeutics, GBM remains challenging to manage, and patients with GBM have very poor prognosis. Compared to lower-grade brain tumors, GBM displays high vasculature due to the high level of hypoxia in its microenvironment [3]. Indeed, heterogeneous regions occur in GBM as a result of oxygen gradients which range from 0.1% to 10% in vivo [4] Furthermore, hypoxia leads to an increase in the messenger RNA (mRNA) levels of the pro-angiogenic inducer, vascular endothelial growth factor A (VEGF) [5,6,7,8]. This occurs through the transactivation of the VEGF promoter via the transcription factor hypoxia-inducible factor (HIF1) [9].

GBM was commonly found to overexpress VEGF [9,10,11,12,13]. In addition, many reports described a correlation between tumor grade and VEGF expression, implicating VEGF in the pathognomonic, histopathologic, and clinical features of GBM tumors in patients [9,10,11,12,14,15]. In fact, inhibiting VEGF through various approaches halts the progression of GBM in vivo and leads to the regression of blood vessels [16,17]. It follows that targeting angiogenesis and specifically VEGF, is a very promising therapeutic approach for managing GBM [18,19]. Hence, understanding signal transduction pathways that regulate VEGF in response to hypoxia in these tumors is exceedingly valuable.

Epidermal growth factor (EGF) signaling is increased in GBM due to gene amplification of the receptor or a mutation in its extracellular domain that renders it constitutively active [20,21,22,23]. EGF signaling is established as an angiogenic inducer positively regulating VEGF production in many cancer types, including GBM [8,15,24,25,26]. A clear interplay between hypoxia and the EGF signal transduction pathway was also described [27,28,29]. For instance, EGF activation of the EGF receptor on GBM cells leads to enhanced secretion of VEGF by GBM cells. EGF signals through the mitogen-activated protein kinase/extracellular signal-regulated kinase (MAPK/ERK) pathway, as well as the phosphatidylinositol 3-kinase (PI3K) pathway in glioblastoma cells and other cell lines [24,30,31,32]. As a result, inhibition of the EGF stimulation of VEGF production by human malignant GBM cells is well established as a model of glioblastoma multiforme pathophysiology [33]. The role of the mitogen-activated protein kinase/extracellular regulated kinase (MEK/ERK) and the PI3K pathways in the regulation of vascular development and angiogenesis in vascular endothelial cells is established [34,35,36,37,38,39]. In addition, both pathways are implicated in angiogenesis in glioblastoma and linked to hypoxia and to VEGF regulation [8,38,40,41,42].

Of the downstream effectors of PI3K, the Rho family of GTPases is the most implicated in angiogenesis [37,43]. The Rho family of GTPases comprises 20 members (in humans) of small GTP-binding proteins with molecular sizes ranging from 20 to 40 kDa [44]. The most studied members are RhoA, RhoC, Rac1, and Cdc42 [45,46,47]. Rho-GTPases acts as switches between active GTP-binding states and inactive GDP-binding states [44]. They get activated by guanine nucleotide exchange factors (GEFs) which are, in many cell types, activated downstream from PI3K [37,48]. GTPase activating proteins (GAPs) and guanine dissociation inhibitors (GDIs) lead to the inactivation of Rho-GTPases [48]. Rho-GTPases are key regulators of the actin cytoskeleton and actin remodeling events required during cellular proliferation and migration [49,50,51]. RhoA, RhoC, Rac1, and Cdc42 regulate endothelial cell proliferation, polarization, cell–cell adhesion, and migration, as well as vascular permeability during angiogenesis [43,52,53,54,55,56,57,58].

Although RhoA and RhoC are established as regulators of angiogenesis [52,58,59,60], most of these studies examined their role on vessel formation in vascular endothelial cells in response to VEGF, rather than their direct role in VEGF production in cancer cells following PI3K activation [60,61,62]. In addition, the interplay between EGF, hypoxia, PI3K, MAPK, and Rho-GTPases during VEGF regulation is yet to be clarified. In this study, we examine the cross-talks between these pathways in response to hypoxia induction by cobalt(II) chloride hexahydrate (CoCl_2_). CoCl_2_ was chosen for its ability to alter the molecular and biochemical targets implicated in hypoxia signaling rather than the level of O_2_ and for sharing many similarities with hypoxia in terms of cell function regulation, including its effects on cell metabolism, on transcription, and on the modulation of key signaling pathways in vivo and in vitro [63].

## 2. Materials and Methods

### 2.1. Cell Culture

Human GBM cell lines SF-268 and U87 were obtained from THE American Type Culture Collection (ATCC, Manassas, VA, USA) and were cultured in Dulbecco’s modified Eagle’s medium (DMEM) supplemented with 10% fetal bovine serum (FBS) and 100 U penicillin/streptomycin at 37 °C and 5% CO_2_ in a humidified atmosphere.

### 2.2. Antibodies and Reagents

Dulbecco’s modified Eagle’s medium (DMEM), fetal bovine serum (FBS), penicillin/streptomycin, bovine serum albumin (BSA), cobalt(II) chloride hexahydrate (CoCl_2_), the PI3K inhibitor wortmannin, the specific MEK1/2 inhibitor U0126, and the specific EGFR inhibitor AG1478 were purchased from Sigma (St Louis, MO, USA). Gibco Leibovitz L-15 medium and recombinant human epidermal growth factor (EGF) were obtained from Invitrogen (Invitrogen, Carlsbad, CA, USA). Hiperfect transfection reagent and human FlexiTube small interfering RNAs (siRNAs) for RhoA (oligo-1 and oligo-6) (NM_001664), RhoC (oligo-1 and oligo-6) (NM_001042678), Cdc42 (NM_001039802), and StarD13 (NM_001243466) were purchased from Qiagen (Qiagen, Germantown, TN, USA). The RhoA/Rac/Cdc42 Activation Assay Combo Kit was obtained from Cell BioLabs (San Diego, CA, USA). The goat polyclonal anti-StarD13 and the rabbit polyclonal anti-Cdc42 (sc-87) antibodies were obtained from Santa Cruz Biotechnology. VEGF-A ELISA kit, rabbit monoclonal anti-VEGF [EP1176y], rabbit monoclonal anti-PTEN, rabbit polyclonal anti-pan-Akt, rabbit polyclonal anti-Akt1 phosphorylated at T308, mouse monoclonal anti-ERK, rabbit polyclonal anti-Erk1 (pT202/pY204) + Erk2 (pT185/pY187), rabbit monoclonal anti-HIF-1α EP1215Y, mouse monoclonal anti-RhoA, rabbit polyclonal anti-RhoC, and rabbit polyclonal anti-beta-actin antibodies were purchased from Abcam (Abcam Inc, Cambridge, UK). Anti-rabbit and anti-mouse HRP-conjugated secondary antibodies were obtained from Promega (Promega, CO., WI, USA). The ECL detection kit used was produced by GE Healthcare (GE Healthcare, Chicago, IL, USA) and the X-ray films were obtained from Agfa (Agfa Healthcare, Greenville, SC, USA).

### 2.3. EGF Stimulation

SF-268 cells were seeded in six-well plates and cultured overnight until they reached 80% confluence. Then, the media were removed and the cells were washed with 2 mL of PBS (1×) before starvation at 37 °C in Leibovitz L-15 medium supplemented with 0.1% BSA. Three hours later, the starvation medium was removed, and the cells were washed with PBS (1×) and stimulated with 15 nM EGF for different time intervals before protein extraction.

### 2.4. Hypoxia Mimicking

SF-268 cells were grown overnight to 80% confluence and stimulated with 200 µM cobalt(II) chloride hexahydrate (CoCl_2_) for the indicated times to induce hypoxia [64]. Subsequently, the cells were washed with ice-cold PBS (1×) and transferred on ice for protein extraction.

### 2.5. Hypoxia Chamber

SF268 cells were plated in Corning 35-mm dishes at a concentration of 2 × 10^4^ cells/mL in complete media. Hypoxia was induced using a STEMCELL technologies hypoxia chamber incubator (Catalog #27310) with mixed gas (1% oxygen, 5% CO_2_, 94% nitrogen). The chamber was purged with hypoxic gas for 15 min to fill the chamber. The cells were kept in the chamber for 24 or 48 h before being lysed.

### 2.6. Inhibitors Treatment

Where indicated, SF-268 cells were pre-treated with U0126 or wortmannin (Wm), to inhibit the ERK and PI3K pathways respectively, in addition to hypoxia mimicking or EGF stimulation. U0126 was used at a final concentration of 50 µM for 24 h, Wm at 100 nM for 1 h, and AG1478 at 10 μM for 24 h. Cells were kept with the drugs during hypoxia mimicking. Prior to EGF stimulation, cells were starved in serum-free media for 3 h, then switched to the EGF stimulation media that contained Wm, where they were incubated for 4 h with Wm and EGF. For U0126 and AG1478 treatments, the drugs were kept throughout the experiment (added to the starvation media and the stimulation media).

### 2.7. Cell Transfection with Small Interfering RNA (siRNA)

The siRNAs used had the following target sequences: RhoA: 5′-CCCGCAATACGCTCAGTTATA-3′, Hs_RhoA:5′-TTCGGAATGATGAGCACACAA-3′, Hs_RhoC:5′–ATGCATTTCCTGGAGAATATA-3′, Hs_StarD13:5′-CCCGCAATACGCTCAGTTATA-3′, and Hs_Cdc42_7: 5′-CATCAGATTTGAAATATTTAA-3′. The cells were transfected with the indicated siRNA at a final concentration of 20 µM using the HiPerfect transfection reagent as per the manufacturer’s recommendations. Control cells were transfected with siRNA sequences targeting GL2 luciferase. After 72 h, the protein levels in total cell lysates were analyzed by Western blot using the appropriate antibodies.

### 2.8. Western Blot Analysis

Whole-cell lysates were prepared by scraping the cells with Laemmli Sample Buffer containing 4% SDS, 20% glycerol, 10% β-mercaptoethanol, 0.004% bromophenol blue, and 0.125 M Tris-HCl (Ph = 6.8). The proteins were then separated by SDS-PAGE under standard and optimized conditions and transferred onto a PVDF membrane. The membranes were then blocked in 5% fat-free milk or 5% BSA for 1 h at room temperature before incubation with the different primary antibodies overnight. The primary antibodies were then removed, and the membranes were washed six times with Tris-buffered saline and 0.1% Tween 20 (TBST) at room temperature. The membranes were then incubated with anti-mouse or anti-rabbit secondary antibodies (1:10,000 or 1:5000 dilution) for 1 h at room temperature. Finally, the blots were developed using the chemiluminescent reagent ECL detection kit, and the bands were visualized on an X-ray film. Protein expression levels were quantified by densitometry analysis using Image J software (1.51J8, NIH, USA).

### 2.9. Pull Down Assays

Pull-down assays were performed using the RhoACdc42/Rac Activation Assay Combo Kit (Cell BioLabs) as previously described [65] and following the manufacturer’s instructions. Briefly, cell lysates were incubated with GST-RBD (Rhotekin binding domain) for 1 h at 4 °C with gentle agitation. Then, the samples were centrifuged, and the pellet was washed several times. After the last wash, the pellets were resuspended with sample buffer and boiled for 5 min. GTP-RhoA was detected by Western blotting using anti-RhoA. Total RhoA was collected prior to the incubation with GST-RBD and used as a loading control. All Western blots were analyzed by ECL (GE Healthcare) followed by densitometry.

### 2.10. ELISA

VEGF-A expression levels in conditioned media were measured using the ELISA sandwich enzymatic method with specific anti-VEGF-A antibodies coated on a 96-well plate according to the manufacturer’s guidelines. Briefly, cancer cells were grown to confluence in media supplemented with 10% FBS before mimicking hypoxia by treatment with cobalt(II) chloride hexahydrate (CoCl_2_). Standards or supernatants from samples were pipetted into the wells containing the immobilized VEGF antibodies. The wells were then washed before adding biotinylated anti-human VEGF antibody. Following incubation, the unbound biotinylated antibody was washed off, and HRP-conjugated streptavidin was pipetted into the wells. After an addition wash step, a TMB substrate solution was added to the wells, resulting in blue coloration proportional to the amount of the bound VEGF. Finally, the stop solution was added, and the colorimetric intensity of the blue substrate now turned yellow was measured at 450 nm.

### 2.11. Statistical Analysis

All results reported represent the average values of at least three independent experiments. Error estimates are given as ± standard error of the mean (SEM). The statistical analysis was performed using the *t*-test, and the statistical significance was set at a *p*-value ≤0.05.

## 3. Results

### 3.1. Hypoxia Mimicking Increases the Expression and Secretion of Vascular Endothelial Growth Factor (VEGF) in GBM Cells

**Figure 1:**
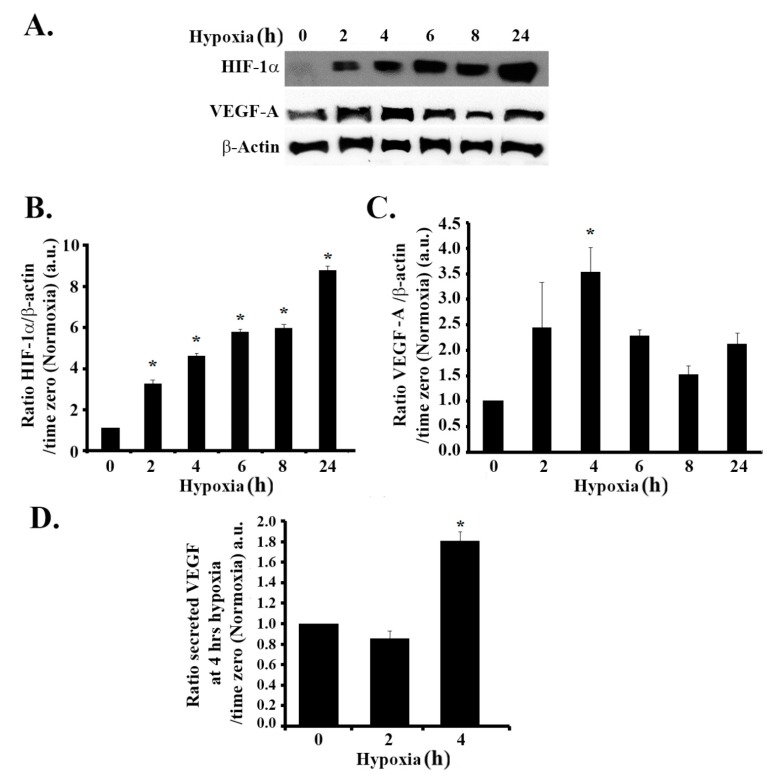
Hypoxia mimicking increases the expression of hypoxia-inducible factor (HIF-1a) and the expression and secretion of vascular endothelial growth factor (VEGF) in glioblastoma cells. (**A**) SF-268 cells subjected to hypoxia using cobalt(II) chloride hexahydrate (CoCl_2_) up to 24 h. The total cell lysates were collected at different time intervals (2, 4, 6, 8, and 24 h post hypoxia or normoxia), as indicated, and the samples were blotted against HIF-1α, VEGF-A, or β-actin antibodies. Quantitation of HIF-1α (**B**) or VEGF-A (**C**) using ImageJ. The bands were normalized to β- actin and expressed as fold change compared to the control (normoxia). (**D**) SF-268 cells were subjected to hypoxia using cobalt(II) chloride hexahydrate (CoCl_2_) for 2 or 4 h or left in normoxic conditions. The supernatant was collected and measured for VEGF-A secretion (compared to standards) according to the manufacturer’s guidelines (described in Section 2). The data are means ± standard error of the mean (SEM) from three different experiments (*n* = 3); * *p* < 0.05 indicates statistically significant differences.

Firstly, we investigated the efficiency of CoCl_2_ in mimicking hypoxia in SF-268 human GBM cells. To this aim, we checked the expression levels of HIF1-α at different time intervals after treatment with CoCl_2_. The results presented in Figure 1A show that HIF-1α expression levels significantly increased in a time-dependent manner as compared to the untreated control. An approximate three-fold increase was seen as early as 2 h post hypoxia, as compared to time zero. The levels of HIF-1α kept accumulating up to 24 h after treatment, suggestive of a hypoxic response (Figure 1A,B).

We then examined the effects of hypoxia on VEGF-A expression levels. In response to CoCl_2_ treatment, the level of VEGF increased by approximately 2.5-fold at 2 h and peaked at 3.5-fold at 4 h, as compared to time zero. The elevation in VEGF-A persisted up to 24 h post treatment (Figure 1A,C). We also detected a significant 1.8-fold increase in VEGF secretion by ELISA 4 h after hypoxia mimicking (Figure 1D).

### 3.2. Hypoxia-Induced Increase in VEGF Expression and Secretion Is ERK-Dependent and PI3K-Independent in GBM Cells

**Figure 2:**
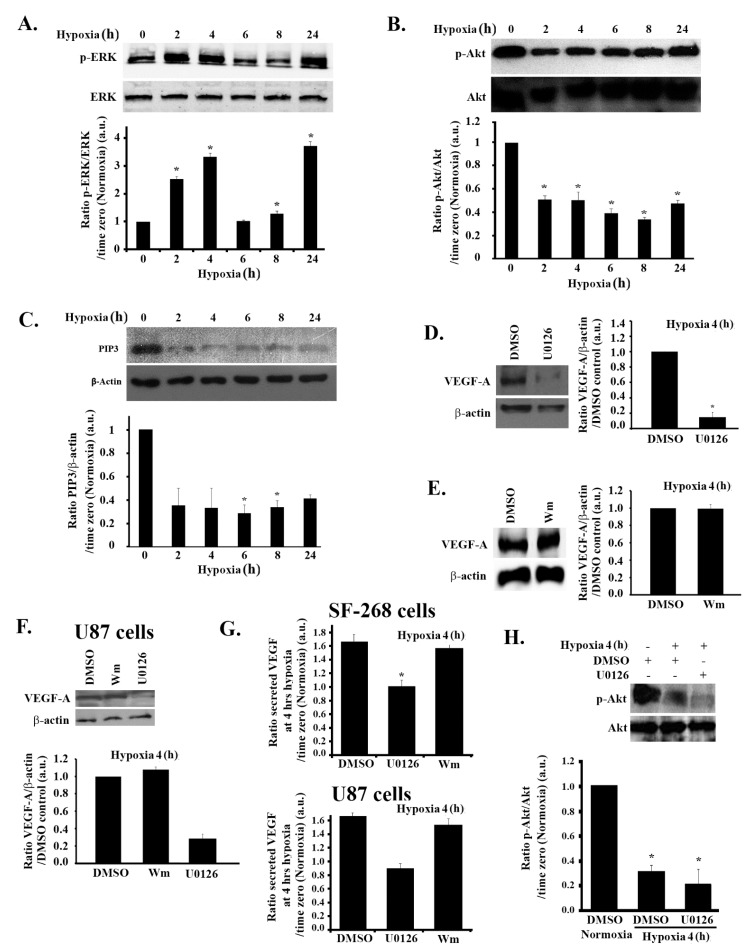
Hypoxia-induced increase in VEGF expression is ERK-dependent but PI3K-independent in glioblastoma cells. (A/B/C) SF-268 cells were subjected to hypoxia using cobalt(II) chloride hexahydrate (CoCl_2_) for the indicated time. Cells were then lysed, and the lysates were blotted for p-ERK and ERK (**A**) and p-Akt and Akt (**B**), as well as PIP_3_ and β-actin for loading control (**C**). The graphs in each panel are densitometric analysis of the Western blots using Image J. Values are normalized to the loading control (ERK, Akt, and β-actin for p-ERK, p-Akt, and PIP_3_, respectively) and expressed as fold change compared to time zero (normoxia). (D/E) SF-268 cells were treated with 50 μM U0126 (with DMSO as a carrier) for 24 h (**D**) or with wortmannin 100 nM (Wm) (with DMSO as a carrier) for 4 h (**E**) or with DMSO as a control. Cells were then subjected to 4 h hypoxia and lysed, and cell lysates were blotted for VEGF-A or β-actin for loading control. The graphs are quantitations for the VEGF bands in (**D/E**) normalized to actin and expressed as fold change compared to control (DMSO). (**F**) U87 cells were treated with 50 μM U0126 for 24 h or with wortmannin 100 nM (Wm) for 4 h (with DMSO as a carrier). Cells were then subjected to 4 h hypoxia and lysed, and cell lysates were blotted for VEGF-A or β-actin for loading control. The graphs are quantitations for the VEGF bands in (**F**) normalized to actin and expressed as fold change compared to control (DMSO). (**G**) ELISA for supernatants from SF-268 cells (upper graph) or U87 cells (lower graph), treated with U0126 or wortmannin or DMSO alone and then kept in normoxia or subjected to 4 h hypoxia. Supernatants were collected and measured for VEGF-A secretion according to the manufacturer’s guidelines. Values are expressed as fold change at every treatment to normoxia. (**H**) (+ indicates addition of the treatment) SF-268 cells were treated with DMSO or with 50 μM U0126 for 24 h, subjected to hypoxia for 4 h. Cells were then lysed, and the lysates were blotted for p-Akt and Akt. The graph is a quantitation of the gels. Values are normalized to Akt and expressed as fold change compared to control (normoxia + DMSO alone). The data are means ± SEM from three different experiments; * *p* < 0.05 indicates statistically significant differences.

The role of the mitogen-activated protein kinase/extracellular signal-regulated kinase (MAPK/ERK) pathway, as well as the phosphatidylinositol 3-kinase (PI3K) pathway, in hypoxia-induced VEGF regulation is well established [24,38]. We next examined the involvement of these pathways in response to hypoxia in GBM. Following the same hypoxia treatment described earlier, we examined ERK phosphorylation kinetics at different times, for up to 24 h after hypoxia induction. As shown in Figure 2A, ERK phosphorylation significantly increased by more than two-fold at 2 h post hypoxia and more than three-fold at 4 h post hypoxia, correlating with the VEGF increase in expression and secretion kinetics.

Surprisingly, however, while examining the kinetics of Akt phosphorylation and Phosphatidylinositol (3,4,5) triphosphate (PIP_3)_ production by Western blot (reflecting the activity of the PI3K pathway), we detected the reverse effect of hypoxia on this pathway. As seen in Figure 2B,C, at 2 h post hypoxia, cells had a significant 50% reduction in p-Akt levels and more than 60% reduction in PIP_3_ levels that persisted for the duration of the treatment, suggesting an inhibition of PI3K by hypoxia.

While an activation of the MAPK pathway and an inactivation of the PI3K pathway correlated with the kinetics of hypoxia-induced VEGF expression and secretion in these cells, it does not prove direct regulation by these pathways. In order to test the direct role of MAPK and PI3K, we used well-established specific inhibitors to inhibit these pathways and directly test the effect on VEGF. Both the PI3K inhibitor wortmannin and the specific MEK1/2 inhibitor U0126 were tested in SF-268 and U87 cells, and their activity was determined through a decrease in p-Akt and p-ERK, respectively, after treatment. Since VEGF expression level and secretion (Figure 1C,D), as well as ERK phosphorylation (Figure 2A), peaked at 4 h post hypoxia, we used this time for these experiments and throughout the rest of the study.

As expected, when the cells were treated with U0126, VEGF expression at 4 h hypoxia was reduced by more than 80% in treated cells compared to control cells (dimethyl sulfoxide, DMSO alone) (Figure 2D).

In line with the observed decrease in the PI3K activity in response to hypoxia, treating cells with the PI3K inhibitor wortmannin did not significantly change the expression levels of VEGF-A, which remained similar to the DMSO control (Figure 2E).

When tested in another GBM cell line (U87), the increase in VEGF-A expression in response to hypoxia was also found to depend on the MAPK but not on the PI3K pathway (Figure 2F). Consistently, secreted VEGF levels in U0126-treated cells were reduced to those of normoxic cells, whereas wortmannin-treated cells did not show a decrease in VEGF secretion in any cell lines (SF-268 and U87 cells), as compared to DMSO-treated cells (Figure 2G).

In addition, since hypoxia mimicking using CoCl_2_ might have slightly different results than those of true hypoxia, we studied the effect of MEK and PI3K inhibition on hypoxia-mediated VEGF expression by incubating SF-268 cells in a hypoxia chamber for 24 or 48 h. The cells showed an increase in HIF levels after 24 h but mostly after 48 h of incubation in the hypoxia chamber (Figure S2A, Supplementary Materials). Consistently, the hypoxia chamber-mediated increase in VEGF expression was found to be inhibited in response to U0126 treatment but not to wortmannin treatment (Figure S2B, Supplementary Materials).

The observed hypoxia-mediated inhibition of PI3K activity (reflected through p-Akt) was also found to be independent of ERK. Treating cells with U0126 did not reverse the decrease in p-Akt seen in hypoxic cells (Figure 2H). This suggests that hypoxia suppressed the PI3K pathway, independently of the MAPK pathway.

### 3.3. The Increase in VEGF Expression in Response to Hypoxia in GBM Cells Is Independent of Rho-GTPases

Although hypoxia-mediated VEGF regulation did not require PI3K, its downstream effector, RhoA, was previously linked to the regulation of VEGF in response to hypoxia in other models [38]. Hence, we sought to examine the involvement of both RhoA and RhoC isoforms in hypoxia-mediated expression and secretion of VEGF in these cells.

**Figure 3:**
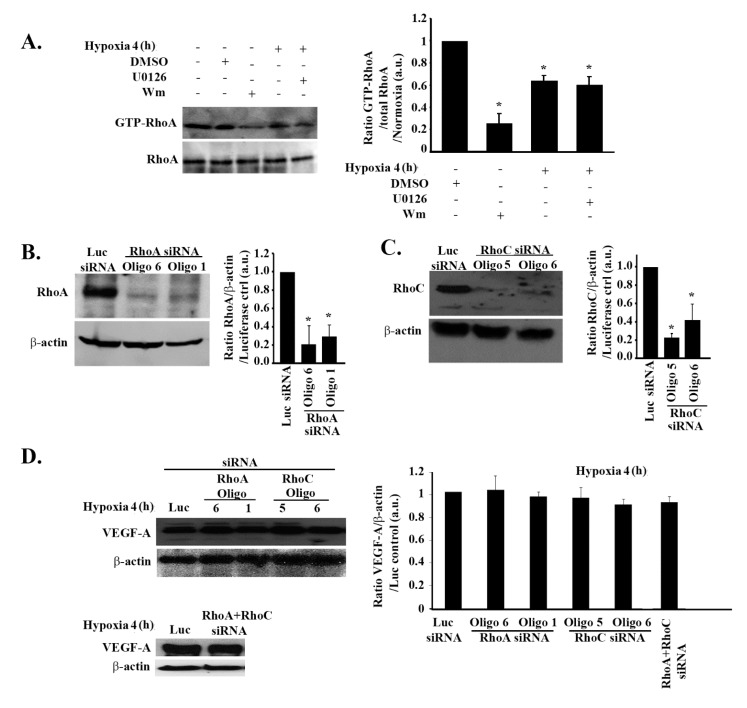
Hypoxia-induced increase in VEGF expression is independent of RhoA and RhoC in glioblastoma cells. (**A**) (+ indicates addition of the treatment) SF-268 cells were treated with DMSO alone or U0126 or Wm, subjected to hypoxia for 4 h, or left in normoxia and lysed. The lysates were then subjected to a RhoA pull-down assay. The upper gel shows the active (GTP-loaded) RhoA that was pulled down with the GST-RBD beads blotted with anti-RhoA, and the lower gel shows the total lysates blotted for anti-RhoA for loading control. The graph is a quantitation of the bands in the upper gel normalized to total RhoA and expressed as fold change compared to time zero (normoxia). (B/C) SF-268 cells were transfected with luciferase siRNA for control of with two RhoA small interfering RNA (siRNA) oligos (oligos 6 and 1) (**B**) or two RhoC siRNA oligos (oligos 5 and 6) (**C**) for 72 h. Cells were then lysed, and cell lysates were blotted for RhoA or β-actin for loading control (**B**) or RhoC and β-actin (**C**). The graphs are quantitations of the bands in (B/C) normalized to actin and expressed as fold change compared to luciferase siRNA control. (**D**) SF-268 cells were transfected with different siRNA oligos as indicated (or doubly transfected with RhoA and RhoC siRNA for double knock down; lower gel), then subjected to 4 h hypoxia or kept in normoxia. Cells were then lysed, and cell lysates were blotted for VEGF-A or β-actin for loading control. The graphs are quantitations of the VEGF bands normalized to actin and expressed as fold change compared to control (luciferase siRNA). The data are means ± SEM from three different experiments (n = 3); * *p* < 0.05 indicates statistically significant differences.

A RhoA pull-down assay analysis confirmed RhoA to be a downstream effector from PI3K in these cells. When cells were treated with wortmannin, the GTP-RhoA level decreased by more than 80% (Figure 3A). The pull-down assay also revealed a significant decrease in RhoA activation in response to hypoxia (4 h), similarly to the decrease in PI3K activity seen in Figure 2. Also, similarly to PI3K activity, RhoA activation was not recovered following U0126 treatment, collectively showing that the hypoxia-mediated inhibition of the PI3K/Rho-GTPase pathway is ERK-independent (Figure 3A).

In addition, no significant change in RhoA or RhoC total protein levels was noted during the 24-h hypoxic time frame (Figure S3, Supplementary Materials). For further analysis, we transfected the cells with RhoA or RhoC siRNA in order to test the direct effect of RhoA or RhoC knock down or the effect of RhoA/RhoC double knock down on VEGF in these cells. The efficiency of the knock down was tested by Western blot. RhoA expression decreased by almost 80% with both oligo 6 and oligo 1 (henceforth, oligo 1 siRNA was used when only one oligo is indicated) and RhoC expression decreased by 80% with oligo 5 and 60% with oligo 6 (henceforth, oligo 5 was used when only one oligo is indicated) (Figure 3B,C).

As seen in Figure 3D, none of the single or double knock downs affected VEGF expression in SF-268 cells subjected to hypoxia, thus ruling out the involvement of the Rho-GTPases in the regulation of VEGF-A expression in response to hypoxia.

### 3.4. The MAPK Pathway Positively Regulates VEGF Expression and Secretion in Response to EGF Stimulation

**Figure 4:**
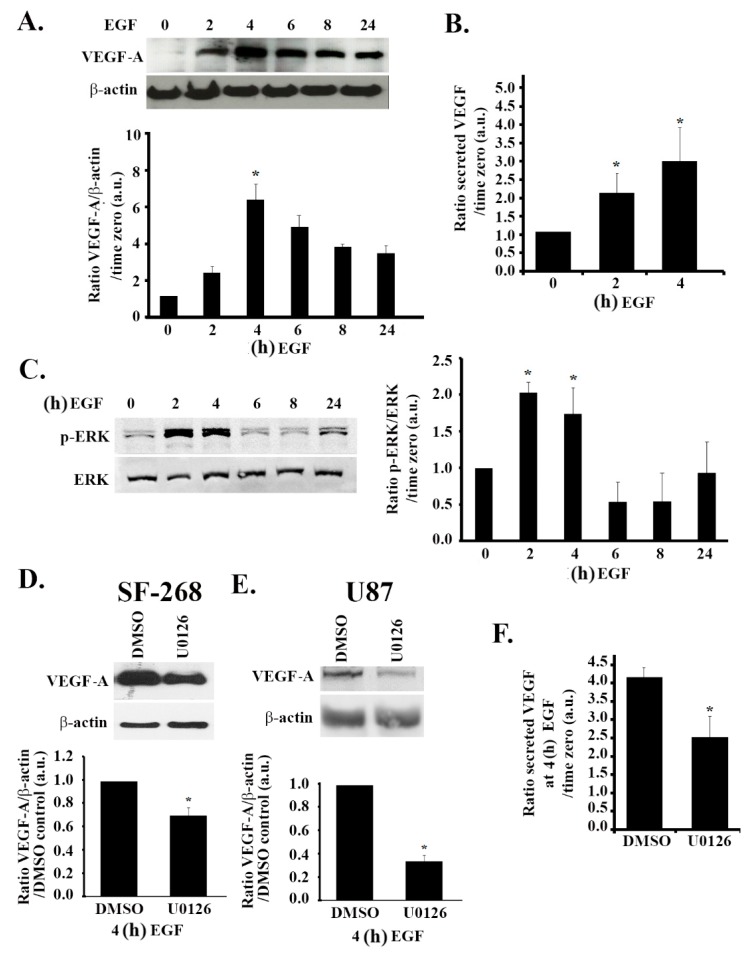
EGF-induced increase in VEGF expression and secretion is ERK-dependent. (A/C) SF-268 cells were starved in serum-free media for 3 h, then stimulated with 15 nM EGF for the indicated times. Cells were then lysed, and the lysates were blotted for VEGF-A and β-actin (**A**) or p-ERK and ERK (**C**). The graphs in each panel are densitometric analysis of the Western blots using Image J. Values are normalized to the loading control (β-actin and ERK for VEGF and p-ERK, respectively) and expressed as fold change compared to time zero (− EGF). (**B**) ELISA for supernatants from SF-268 cells treated with EGF for 2 or 4 h or left untreated and measured for VEGF-A secretion according to the manufacturer’s guidelines. Values are expressed as fold change compared to time zero. (**D**) SF-268 cells were treated with 50 μM U0126 for 24 h (with DMSO as carrier). Cells were then treated with 15 nM EGF for 4 h and lysed, and cell lysates were blotted for VEGF-A or β-actin for loading control. The graphs are quantitations of the VEGF bands normalized to actin and expressed as fold change compared to control (DMSO). **E)** U87 cells were treated with 50 μM U0126 for 24 h (with DMSO as a carrier). Cells were then treated with 15 nM EGF for 4 h and lysed, and cell lysates were blotted for VEGF-A or β-actin for loading control. The graphs are quantitations of the VEGF bands normalized to actin and expressed as fold change compared to control (DMSO). (**F**) Supernatants from SF-268 cells treated with U0126 or DMSO alone and then treated with EGF for 4 h (after 3 h starvation) were collected and measured for VEGF-A secretion according to the manufacturer’s guidelines. Values are expressed as fold change at every treatment to time zero (− EGF). The data are means ± SEM from three different experiments; * *p* < 0.05 indicates statistically significant differences.

In human glioblastoma cells, the epidermal growth factor receptor (EGFR) is implicated as a downstream mediator of hypoxic effects [33]. Indeed, treating SF-268 cells with EGF (after starvation in serum-free media) mimicked the effect of hypoxia on VEGF expression, as well as secretion in these cells. We, therefore, examined the effects of EGF treatment on VEGF expression levels. The data in Figure 4A,B show an increase in the expression levels and secretion levels of VEGF-A by approximately six-fold and three-fold, respectively, in response to stimulation with 15 nM EGF, as compared to the untreated control. Similarly to hypoxia, EGF stimulation also led to an increase in ERK phosphorylation (two-fold) (Figure 4C). It is noteworthy that the kinetics of the increase in expression of VEGF-A and the increase in ERK phosphorylation were similar to those observed following hypoxia, with a peak in expression/phosphorylation at 4 h post treatment and a decrease after 6 h (Figure 4A,C compared to Figure 1C and Figure 2A). In addition, when the cells were treated with U0126, the EGF-induced VEGF-A expression and secretion were reduced by a significant 30% and 40%, respectively, at 24 h after EGF stimulation, thus confirming the role of the MAPK pathway in VEGF regulation following EGF stimulation (Figure 4D,F). Consistently, a significant decrease in EGF-induced VEGF-A expression was seen in U87 cells treated with U0126 (Figure 4E).

### 3.5. The PI3K Pathway Is also Involved in EGF Stimulation-Mediated VEGF Expression and Secretion

**Figure 5:**
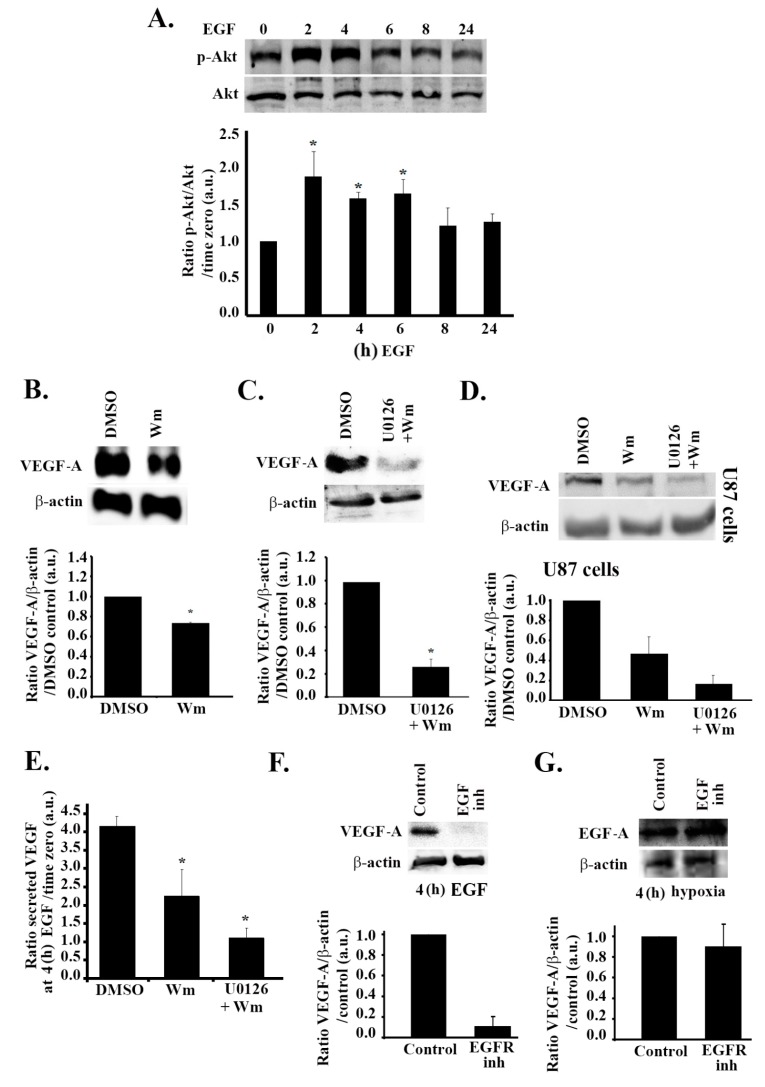
EGF stimulation leads to an increase in VEGF expression and secretion through PI3K and ERK in parallel to hypoxia. (**A**) SF-268 cells were starved in serum-free media for 3 h, then stimulated with 15 nM EGF for the indicated times. Cells were then lysed, and the lysates were blotted for p-Akt and Akt. The graph is a densitometric analysis of the Western blots using Image J. Values are normalized to Akt and expressed as fold change compared to time zero (− EGF). (B/C) SF-268 cells were treated with either DMSO alone or Wm (100 nM) (**B**) or with DMSO alone or U0126 (50 μM) + Wm (100 nM) (**C**). Cells were then treated with 15 nM EGF for 4 h (treatment detailed in Section 2) and lysed, and cell lysates were blotted for VEGF-A or β-actin for loading control. The graphs are quantitations of the VEGF bands normalized to actin and expressed as fold change compared to control (DMSO). (**D**) U87 cells were treated with either DMSO alone or Wm (100 nM) or U0126 (50 μM) + Wm (100 nM), treated with 15 nM EGF for 4 h (treatment detailed in Section 2), and lysed, and cell lysates were blotted for VEGF-A or β-actin for loading control. The graphs are quantitations of the VEGF bands normalized to actin and expressed as fold change compared to control (DMSO). (**E**) Supernatants from SF-268 cells treated with DMSO alone, Wm alone, or Wm + U0126 and then treated with EGF for 4 h (after 3 h starvation) were collected and measured for VEGF-A secretion according to the manufacturer’s guidelines. Values are expressed as fold change at every treatment to time zero (− EGF). (**F**) SF-268 cells were treated with 10 μM EGFR inhibitor AG1478 or left untreated, and then treated with EGF for 4 h. Cells were then lysed and blotted with anti-VEGF-A and anti-β-actin. The graph is a quantitation where the values were normalized to actin and expressed as fold change compared to control (− AG1478). (**G**) SF-268 cells were treated with 10 μM EGFR inhibitor AG1478 or left untreated and then subjected to 4 h hypoxia, or left in normoxia. Cells were then lysed and blotted with anti-VEGF-A and anti-β-actin. The graph is a quantitation where the values were normalized to actin and expressed as fold change compared to control (− AG1478). The data are means ± SEM from three different experiments; * *p* < 0.05 indicates statistically significant differences.

To further test whether the EGF pathway mediates the effect of hypoxia in these cells, we investigated its effect on the PI3K pathway. To this aim, we assessed the level of phosphorylation of Akt at different time points after treatment with EGF. To our surprise, there was a significant increase in Akt phosphorylation in response to EGF stimulation as compared to the untreated control. Quantitatively, this increase was approximately two-fold following 2 h of treatment with EGF and was sustained with an approximate increase of 1.6-fold at 4 h of treatment (Figure 5A). In addition, when the cells were treated with wortmannin, which inhibits Akt phosphorylation, VEGF expression was reduced by 40%, thus confirming the implication of the PI3K pathway in VEGF expression upon stimulation with EGF (Figure 5B). In order to ensure that the differential effect of PI3K in hypoxia as compared to EGF stimulation is not due to differences in other experimental conditions (serum presence in the hypoxia experiment versus serum removal before EGF stimulation), cells were treated with the inhibitors, subjected to hypoxia, and starved before being blotted for VEGF (Figure S4, Supplementary Materials). The results seen were consistent with Figure 2. This suggests that hypoxia leads to an increase in VEGF expression and secretion in glioblastoma cells through the MAPK/ERK pathway and independently of the PI3K pathway.

When the cells were treated with U0126 alone or wortmannin alone, the EGF-mediated increase in VEGF-A expression or secretion was only partially reduced (Figure 4D and Figure 5B). However, when the cells were treated with both inhibitors, an 80% decrease in VEGF-A levels was observed, as compared to controls (Figure 5C), reaching levels of VEGF secretion comparable to those seen in normoxia (Figure 5E). The same was observed in U87 cells (Figure 5D), where a combination of U0126 and wortmannin treatment led to an added inhibitory effect on VEGF expression. This shows that both ERK and PI3K are involved in the regulation of VEGF expression and secretion downstream from EGF signaling.

### 3.6. Hypoxia and the EGF Receptor Signal in Parallel Leading to VEGF Expression in Glioblastoma Cells

In order to test if hypoxia mediates its effect through the EGF receptor, SF-268 cells were treated with the EGF receptor inhibitor AG1478, or left untreated, and then subjected to hypoxia and tested for VEGF-A expression. Figure 5F,G show that, while treatment with AG1478 abolished the EGF-mediated increase in VEGF, the hypoxia-mediated increase in VEGF levels was not affected by the inhibition of the EGF receptor. This suggests that hypoxia and the EGF receptor lead to an increase in VEGF expression and secretion through separate pathways.

### 3.7. RhoA and RhoC Mediate PI3K-Regulated Increase in VEGF Expression and Secretion in Response to EGF Stimulation in Glioblastoma Cells

**Figure 6:**
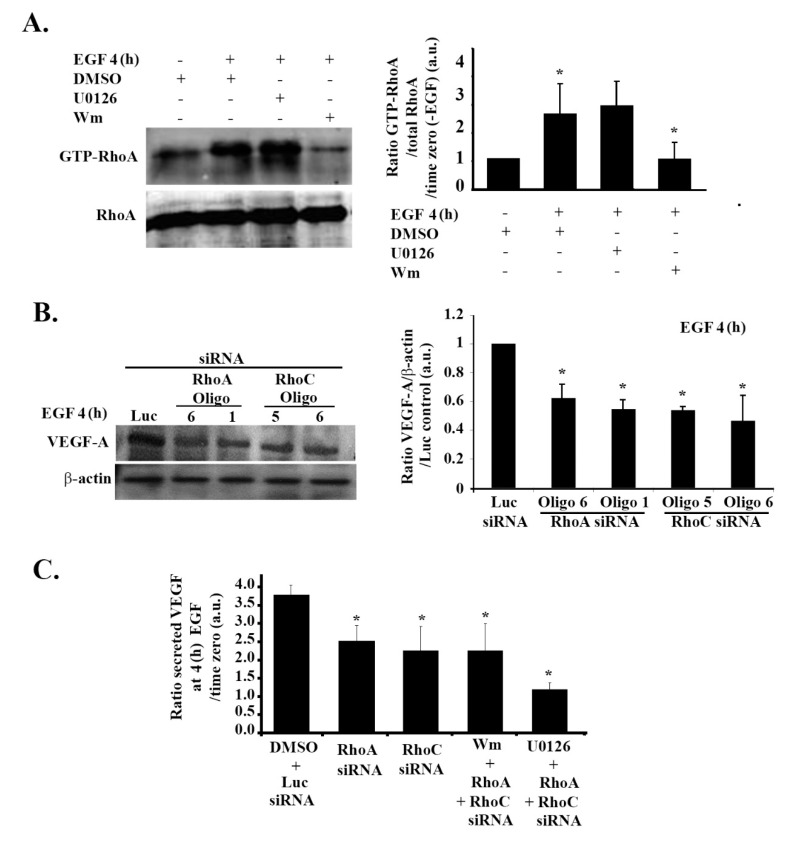
RhoA and RhoC mediate PI3K-regulated increase in VEGF expression and secretion in response to EGF stimulation in glioblastoma cells. (**A**) (+ indicates addition of the treatment) SF-268 cells were starved in serum-free media, then stimulated with 15 nM EGF for 4 h and treated with DMSO alone, U0126 (50 μM), or Wm (100 nM) (treatment sequence explained in Section 2). The lysates were then subjected to a RhoA pull-down assay. The upper gel shows the active (GTP-loaded) RhoA that was pulled down with the GST-RBD beads blotted with anti-RhoA, and the lower gel shows the total lysates blotted for anti-RhoA for loading control. The graph is a quantitation of the bands in the upper gel normalized to total RhoA and expressed as fold change compared to time zero (− EGF). (**B**) SF-268 cells were transfected with different siRNA oligos as indicated, starved, and stimulated with EGF for 4 h. Cells were then lysed, and cell lysates were blotted for VEGF-A or β-actin for loading control. The graphs are quantitations of the VEGF bands normalized to actin and expressed as fold change compared to control (luciferase siRNA). (**C**) ELISA for supernatants from SF-268 cells transfected with the indicated oligos, treated with DMSO, Wm, or U0126, then starved and stimulated with EGF for 4 h or left unstimulated and measured for VEGF-A secretion. Values are expressed as fold change compared to time zero (− EGF). The data are means ± SEM from three different experiments (*n* = 3); * *p* < 0.05 indicates statistically significant differences.

Contrary to the decrease in RhoA activation under hypoxic conditions (Figure 3A), cells treated with EGF for 4 h had an increase in RhoA activation that was reversed in cells treated with wortmannin, but not in cells treated with U0126 (Figure 6A). This confirmed the PI3K-dependent increase in RhoA activation in response to EGF stimulation in these cells. When cells were transfected with either RhoA or RhoC siRNA, a significant decrease in the EGF-induced VEGF expression was observed (Figure 6B). VEGF secretion was also significantly reduced in response to RhoA/C knock down (Figure 5C). Expectedly, knocking down RhoA/C on its own or adding wortmannin to the knock-down cells did not show any added inhibition to VEGF levels. This and data in Figure 5A confirm that RhoA and RhoC signal downstream from PI3K, leading to VEGF expression and secretion in these cells. In addition, while the RhoA/C knock-down ± wortmannin samples showed a partial effect on VEGF, similar to the wortmannin alone treatment (as seen in Figure 5D), combining the siRNA oligos with U0126 led to a decrease in VEGF secretion level back to the normoxic levels (Figure 6C). This was also similar to the amplitude of inhibition seen with wortmannin and U0126 (Figure 5C,D), proving again that the PI3K/RhoA/C pathway and the ERK pathway work in parallel and have an additive effect on VEGF regulation.

### 3.8. Hypoxia Suppresses RhoA/C through StarD13

**Figure 7:**
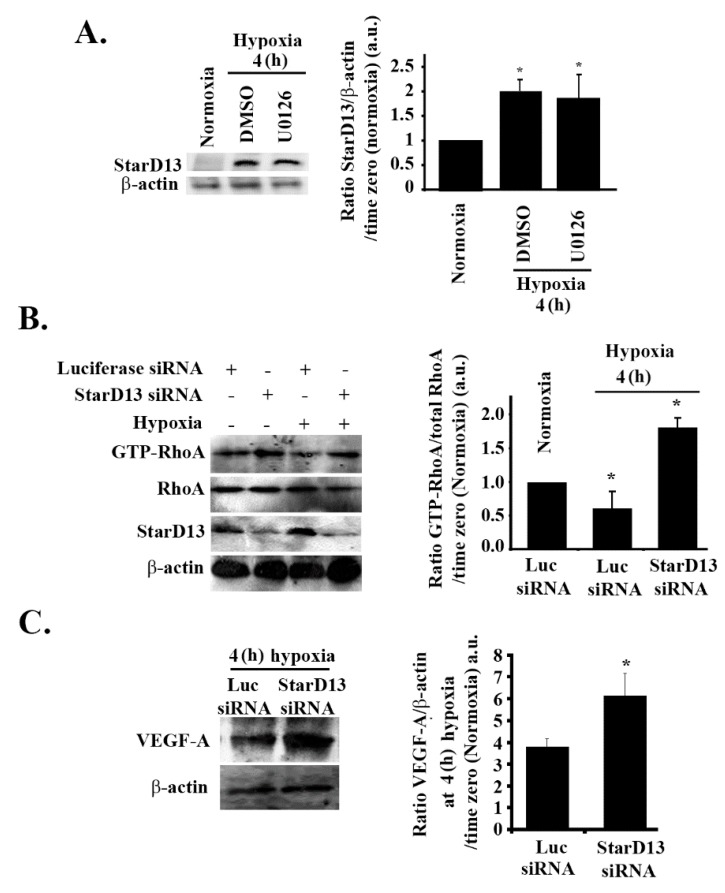
Hypoxia suppresses RhoA/C through StarD13. (**A**) SF-268 cells were treated with either DMSO or U0126 and subjected to hypoxia for 4 h or kept in normoxia. Cells were then lysed, and the lysates were blotted for StarD13 and β-actin for loading control. The graph is a densitometric analysis of the Western blots using Image J. Values are normalized to the loading control and expressed as fold change compared to time zero (normoxia). (**B**) (+ indicates addition of the treatment) SF-268 cells were transfected with either luciferase or StarD13 siRNA and subjected to hypoxia for 4 h, or left in normoxia and lysed. The lysates were then subjected to a RhoA pull-down assay, and the total lysates were blotted for anti-RhoA, anti-StarD13, and anti-β-actin. The graph is a quantitation of the bands in the upper gel normalized to total RhoA and expressed as fold change compared to control (luciferase siRNA/normoxia). (**C**) SF-268 cells transfected with luciferase or StarD13 siRNA and subjected to 4 h hypoxia. Cells were then lysed, and cell lysates were blotted for VEGF-A or β-actin. The graphs are quantitations of the VEGF bands normalized to actin and expressed as fold change compared to normoxia control. Values are expressed as fold change at every treatment to normoxia. The data are means ± SEM from three different experiments (*n* = 3); * *p* < 0.05 indicates statistically significant differences.

In previous studies, we established the tumor suppressor StarD13 (steroidogenic acute regulatory protein)-related lipid transfer domain 13) as a GTPase-activating protein (GAP) that specifically inhibits RhoA and Cdc42 in GBM cells and other tumor models [66,67,68]. We examined the possibility of the hypoxia-induced inhibition of RhoA being mediated through StarD13. Cells subjected to hypoxia for 4 h showed a two-fold increase in the level of StarD13 proteins in SF-268 cells (Figure 7A). This increase was not reversed with U0126 treatment, suggesting that hypoxia directly modulates StarD13 expression. A Western blot analysis and an RhoA pull-down assay confirmed the efficiency of StarD13 knock down, the increase in RhoA activation in StarD13 knock-down cells in normoxia, and the recovery of RhoA activation in hypoxic conditions after StarD13 knock down (Figure 7B). This confirmed that the inhibition of RhoA by hypoxia in these cells is mediated by StarD13. Finally Figure 7C shows that, in response to hypoxia, the recovery of RhoA activation (through StarD13 knock down and the relief of inhibition of RhoA), led to a substantial increase in VEGF expression.

Although StarD13 is a GAP for both RhoA and Cdc42 in these cells, as shown before [66] and as tested again here (Figure S5A, Supplementary Materials), the involvement of Cdc42 in VEGF regulation was ruled out here. This was based on data shown in Figure S5B–D (Supplementary Materials) where Cdc42 activation was not changed under hypoxic conditions in these cells, and knocking down Cdc42 in cells treated with EGF or subjected to hypoxia did not have an effect on VEGF levels.

## 4. Discussion

The angiogenic mechanism of action of VEGF in endothelial cells was extensively described in the literature [19,69,70]. This work investigates different pathways and signaling molecules involved in VEGF production in response to hypoxia in GBM cells, including the MAPK/ERK pathway and the PI3K/RhoA/C pathway. Hypoxia mimicking successfully led to an increase in HIF-1α and VEGF in SF-268 cells. While VEGF showed trace amounts under normoxia, HIF-1α was completely absent from the cell lysates under normoxic conditions. HIF-1 is made up of two components: HIF-1α and Aryl hydrocarbon receptor nuclear translocator (ARNT). The latter is continuously expressed, while HIF-1α stabilizes under hypoxic conditions [71]. This explains why HIF-1α is not detected in GBM cells under normoxic conditions, but significantly increases under hypoxic conditions simulated by CoCl_2_, a chemical inducer of HIF-1α [72]. The increase in HIF-1α triggers the expression of a number of genes including erythropoietin, various glycolytic enzymes, surviving, and VEGF [38,64,72,73]. Our results demonstrate a build-up of HIF-1α following hypoxia, as well as an increase in VEGF expression, thus highlighting the role played by hypoxia in the regulation of VEGF expression in cancer and, more specifically, in glioblastoma [7,38].

Studying the kinetics of ERK phosphorylation in response to hypoxia and EGF stimulation showed an increase preceding and coinciding with the increase in VEGF expression observed at 4 h. This led us to hypothesize a possible involvement of the MAPK pathway in VEGF expression. This hypothesis was supported by the decrease in VEGF expression as compared to control in response to treatment with the ERK inhibitor. In hypoxia, however, both Akt and PIP_3_ phosphorylation decreased with time, suggesting an inhibition of PI3K in hypoxic conditions. One possible explanation could be that hypoxia switches the cell dependence from both PI3K and MAPK pathways to the MAPK pathway alone, in order to suppress cell proliferation, leading to cell entry into a quiescent state until the oxygen supply replenishes [74]. This can also be attributed to the differences in mechanisms of induction of HIF-1α by true hypoxia versus CoCl_2_. Specifically, this is consistent with findings showing that HIF-1α expression upon treatment of He-La cells with CoCl_2_ is dependent on both the MAPK and PI3K pathways, and inhibited by their respective inhibitors, whereas true hypoxia induces HIF-1α in a transient rather than sustained manner and is independent of the aforementioned pathways [75]. Furthermore, VEGF expression at 4 h under hypoxia was not affected by treatment with the PI3K inhibitor wortmannin. This is in line with the findings of Tejado et al., who showed that various PI3K inhibitors including wortmannin do not have an effect on the expression levels of HIF-1α or its transcriptional activity [76]. It was noteworthy that an increase in p-ERK and in p-Akt was evident at 24 h after persistent hypoxia. This is reminiscent of the involvement of these pathways in acute and in chronic hypoxia [77]. It could also be interpreted as a potential pro-apoptotic role of these pathways after persistent hypoxia, which was previously described [78].

In human glioblastoma cells, the epidermal growth factor receptor (EGFR) is implicated as a downstream mediator of hypoxic effects [33]. Indeed, treating SF-268 cells with EGF (after starvation in serum-free media) mimicked the effect of hypoxia on VEGF expression and secretion in these cells. However, EGF stimulation led to an increase in both MAPK and PI3K pathway activation, with both pathways contributing to the increase in VEGF expression and secretion as demonstrated by the partial reversal of this increase in cells treated with either the MAPK of the PI3K inhibitor. However, the effect of using both inhibitors was additive, suggesting that MAPK and PI3K are not in the same pathway downstream from the EGF receptor (similar to lack of U0126 effect on p-Akt in response to hypoxia in Figure 2F) and that the two pathways signal in parallel and do not compensate for one another. In line with previous studies, we demonstrated that both PI3K and MAPK pathways regulate VEGF expression and secretion in response to EGF stimulation in GBM [22,38,39]. However, we show that ERK inhibition leads to an almost complete loss of hypoxia-induced VEGF expression (Figure 2D), as compared to only a 30% decrease in EGF-induced VEGF expression. This suggests that ERK is necessary and sufficient for the expression and secretion of VEGF in hypoxic conditions in these cells. The data in Figure 4 also reveal a more robust increase in VEGF-α expression, as well as secretion, in response to EGF stimulation (six-fold and three-fold, respectively), as compared to hypoxia (3.5-fold and 1.8-fold, respectively) at 4 h (Figure 1). This further suggests the additive effect of the MAPK pathway and the PI3K pathway, leading to VEGF expression in these cells.

Collectively the data above suggest that either (1) a parallel signaling of hypoxia and the EGF receptor exists, leading to an increase in VEGF expression, or (2) hypoxia signals through the EGF receptor, which activates both MAPK and PI3K pathways in normoxia, but suppresses the PI3K pathway, switching to a strictly MAPK-dependent VEGF expression. In order to elucidate which hypothesis is applicable in these cells, we inhibited the EGF receptor in hypoxia-treated cells. We found that the hypoxia-mediated increase in VEGF levels was not affected by the inhibition of the EGF receptor, suggesting that hypoxia and the EGF receptor lead to an increase in VEGF expression and secretion through separate pathways. Hypoxia only activates ERK, which accounts for a more modest increase in VEGF levels, while EGF stimulation activates both PI3K and ERK, which cooperate, resulting in a more pronounced increase in VEGF levels. These findings are in line with previous reports that describe a hypoxia-independent VEGF regulation by EGF and PI3K and conclude that hypoxia could interact with EGF stimulation to increase VEGF levels in an additive manner [8]. Interestingly, the gels in Figure 4A,C reveal the complete lack of VEGF-α and p-ERK (in absolute values) in starved cells (serum-free media) before EGF stimulation as opposed to their basal presence in cells in Figure 1A and Figure 2A in normoxic conditions. This can be explained by the presence of trace amounts of EGF in the serum in those cells.

Contrary to the decrease in RhoA activation under hypoxic conditions (Figure 3A), cells treated with EGF for 4 h showed an increase in RhoA activation that was reversed in cells treated with wortmannin but not in cells treated with U0126 (Figure 5A). This confirmed the PI3K-dependent increase in RhoA activation in response to EGF stimulation in these cells. Transfection of these cells with either RhoA or RhoC siRNA led to a significant decrease in the EGF-induced VEGF expression (Figure 6B). VEGF secretion was also significantly reduced in response to RhoA/C knock down (Figure 5C). Expectedly, knocking down RhoA/C on its own or adding wortmannin to the knock-down cells did not have an added inhibition to VEGF levels. This and data in Figure 5A confirm that RhoA and RhoC signal downstream from PI3K, leading to VEGF expression and secretion in these cells. In addition, while the RhoA/C knock-down ± wortmannin samples showed a partial effect on VEGF, similar to the wortmannin alone treatment (as seen in Figure 5D), combining the siRNA oligos with U0126 led to a decrease in VEGF secretion level back to the normoxic levels (Figure 6C). This was also similar to the amplitude of inhibition seen with wortmannin and U0126 (Figure 5C,D), proving again that the PI3K/RhoA/C pathway and the ERK pathway work in parallel and have an additive effect on VEGF regulation.

We also observed that, while Rho-GTPases are suppressed under hypoxic conditions, independently from ERK, they are activated in response to EGF stimulation, downstream of PI3K. This was in line with similar results in the literature which link the stimulation of the EGFR to the activation of the PI3K pathway and the downstream Rho-GTPases necessary for actin polymerization and motility [32,79,80]. In addition, the Rho-GTPases, as well as the PI3K pathway, take part, along with MAPK pathway, in increasing VEGF expression 4 h after EGF stimulation. This was evident by the significant decrease in the expression levels of VEGF following treatment with RhoA and RhoC knock downs and in the additive effect of VEGF decrease when U0126 was added to the siRNA. This shows that, similarly to PI3K, the Rho-GTPases act in a parallel pathway to that of ERK, leading to VEGF expression, and that both pathways are additive. Interestingly, our results also show that RhoA and RhoC are not redundant and do not compensate for one another. Knocking down either shows that both are required for VEGF expression. Altogether these data allow us to propose a differential mode for VEGF-A expression in response to hypoxia mimicking or EGF stimulation. Hypoxia increases VEGF expression through the MAPK pathway, while suppressing the PI3K and Rho-GTPases pathways. The mechanism of suppression of the PI3K pathway is yet to be determined; however, we assume that the Rho-GTPases are suppressed by the MAPKs. However, this suppression is reversed upon EGF stimulation, whereby the PI3K pathway and Rho-GTPases become involved in VEGF expression along with the MAPKs.

StarD13 (START-GAP2) is a GTPase-activating protein (GAP) that specifically inhibits RhoA and Cdc42 in glioblastoma cells and other tumor models [66,67,68]. When examining a potential hypoxia-induced inhibition of RhoA through StarD13, we found that cells in hypoxic conditions had an increase in StarD13 levels (which was not reversed with U0126 treatment). Collectively with data seen in Figure 2F and Figure 6A, this showed that hypoxia inhibits the PI3K pathway and Rho-GTPases, in parallel to activating the MAPK pathway (since U0126 treatment did not reverse the effect of hypoxia on p-Akt decrease, RhoA activation decrease, or StarD13 level increase). Finally, Figure 7C showed that, in response to hypoxia, the recovery of RhoA activation (through StarD13 knock down and the relief of inhibition of RhoA) led to a substantial increase in VEGF expression, the amplitude of which was comparable to the increase seen in response to EGF stimulation (Figure 4A). This suggests that StarD13 is sufficient to attenuate the regulatory effect of hypoxia on VEGF levels in these cells.

**Figure 8:**
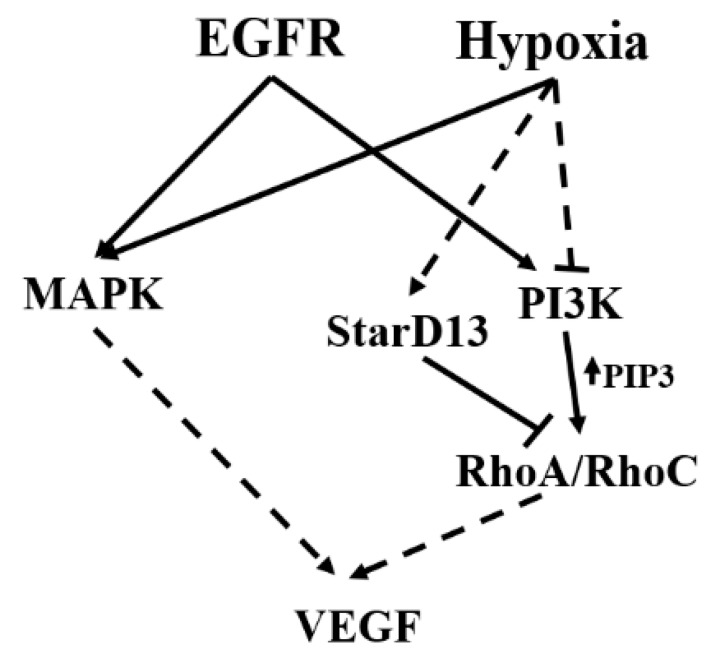
Model showing the pathways involved in VEGF-A expression and secretion in brain tumor cells following hypoxia and EGF stimulation. Hypoxia leads to an increase in VEGF expression and secretion in glioblastoma cells. This is through the EGF receptor and the activation of the MAPK pathway. Hypoxia also suppresses the PI3K/Rho-GTPase pathway, potentially through an increase in the expression of the RhoA GAP, StarD13.

In conclusion, this study shows that, under normoxic conditions, EGF stimulates the activation of both the PI3K and the MAPK pathways and the induction of VEGF in GBM cells. However, hypoxic conditions lead to the suppression of the PI3K/RhoA/C pathway, in part through StarD13, and a switch to a MAPK pathway-dependent VEGF expression only (see Figure 8 for a model). Further work is needed in order to elucidate the mechanism of inhibition of the PI3K pathway by hypoxia.

## Supplementary Materials

The following are available online at https://www.mdpi.com/2073-4409/8/11/1397/s1. Figure S1: Wortmanin and U0126 proof of inhibition of PI3K and MEK1/2, respectively, in SF-268 and U87 cells. Figure S2: Different hypoxic conditions. Figure S3: Hypoxia does not affect the expression of RhoA or RhoC in astrocytoma cells. Figure S4: Hypoxia-induced increase in VEGF expression is ERK-dependent but PI3K-independent even in the absence of serum in astrocytoma cells. Figure S5: Hypoxia does not affect the activation of Cdc42 in astrocytoma cells.
