# Supplementary material for: Hypoxia and EGF Stimulation Regulate VEGF Expression in Human Glioblastoma Multiforme (GBM) Cells by Differential Regulation of the PI3K/Rho-GTPase and MAPK Pathways
Source: Cells. 2019 Nov 6;8(11):1397. doi: 10.3390/cells8111397 (PMC6912653; doi:10.3390/cells8111397)
Supplement: Supplementary file 1 [file cells-08-01397-s001.pdf]

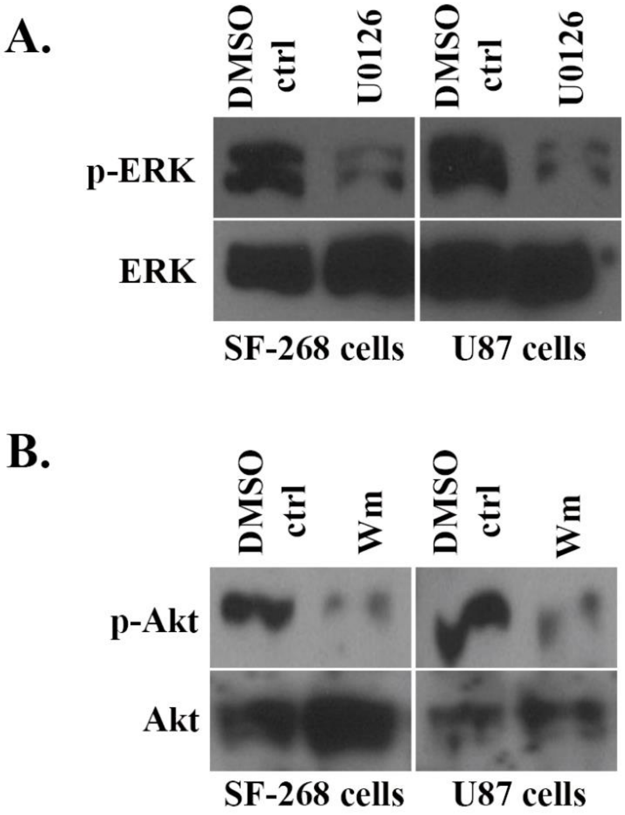

Supplemental Figure S1: Wortmanin and U0126 proof of inhibition of PI3K and MEK1/2, respectively, in SF-268 and U87 cells. SF-268 cells or U87 cells (as indicated) were left in serum, treated with either DMSO alone, with U0126 at a final concentration of 50 μM for 24 h **(A),** or with Wm at 100 nM for 1 h **(B).** Cells were then lysed and Western blots were performed to total lysates blotting for p-ERK and total ERK **(A)** or for p-Akt and total Akt **(B).**

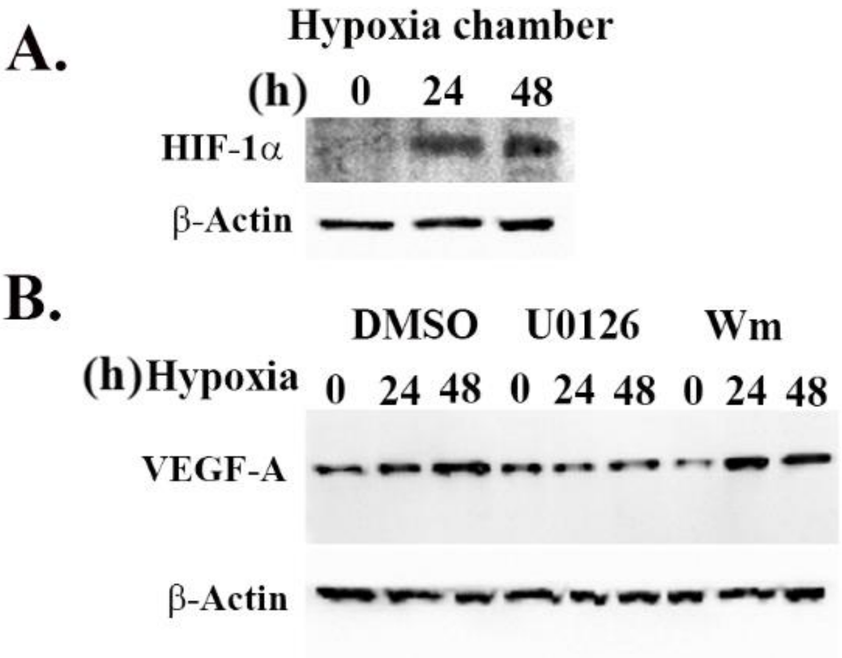

Supplemental Figure S2: Different hypoxic conditions. **(A)** and **(B)** SF268 cells were plated, Hypoxia was then induced using STEMCELL technologies hypoxia chamber incubator with mixed gas (1% Oxygen, 5% CO2, 94% Nitrogen). The chamber was purged with hypoxic gas for 15 minutes to fill the chamber. The cells were subjected to 1. Normoxia (5% CO2 incubator), 2. Hypoxia for 24 hours, or 3. Hypoxia for 48 hours, lysed and blotted for HIF and for actin (A). In (B), the cells were kept in Normoxia for 24 h or subjected to hypoxia for 24 or 48 h. Cells were then treated with U0126 in hypoxia or with wortmanin for 1 h (last hour of the 24 or 48 h hypoxia period). Cells were then lysed and blotted for VEGF and actin.

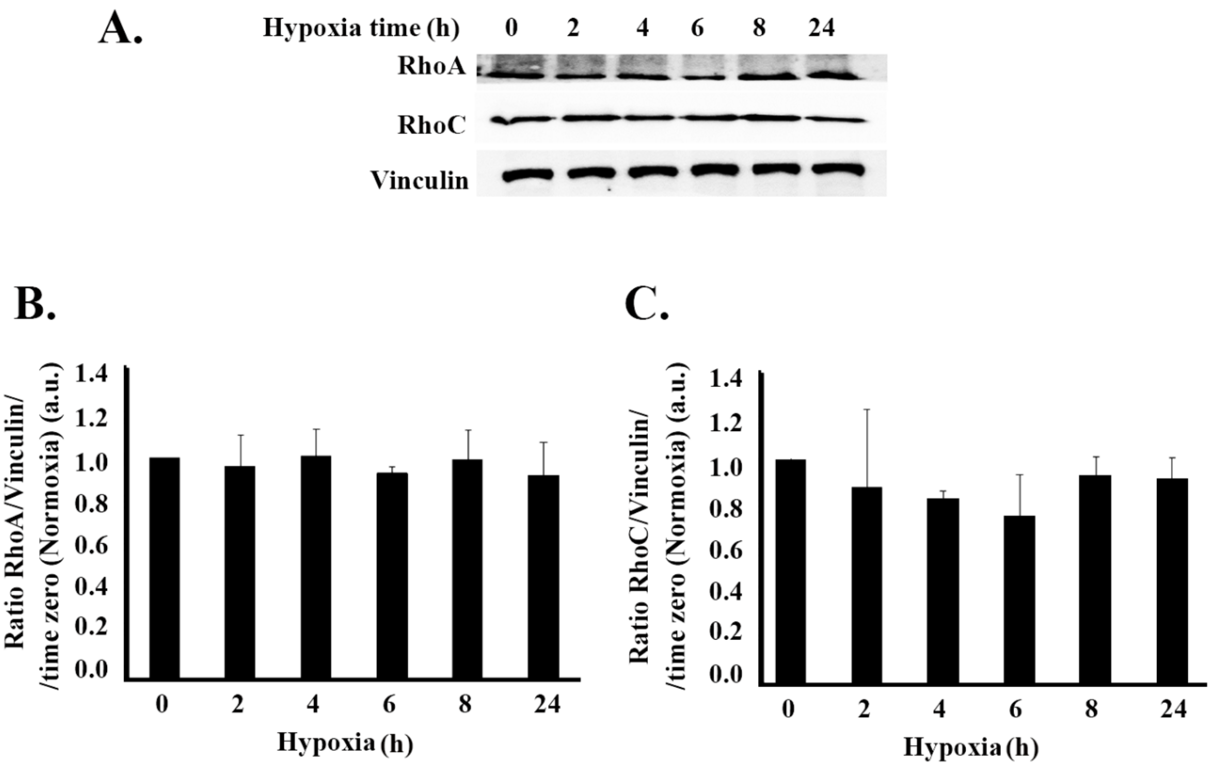

Supplemental Figure S3: Hypoxia does not affect the expression of RhoA or RhoC in astrocytoma cells. **(A)** SF-268 cells were subjected to hypoxia for the indicated times (or left at normoxia), cells were then lysed and lysates blotted against RhoA, RhoC or vinculin as a loading control. (B/C) Quantitation of bands in (A) for the levels of RhoA (B) or RhoC (C) normalized to vinculin and expressed as fold change to time zero (normoxia). The data are the mean ± SEM from 3 different experiments (n=3). *P<0.05 indicates statistically significant differences.

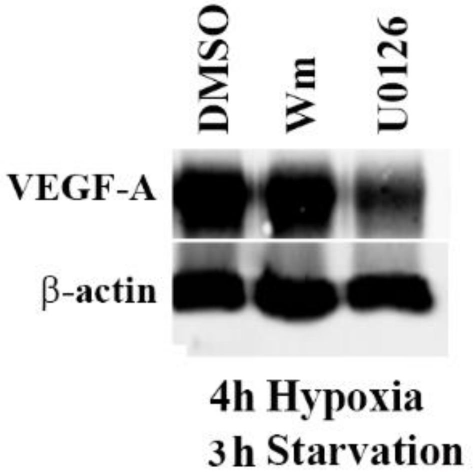

Supplemental Figure S4: Hypoxia-induced increase in VEGF expression is ERK-dependent but PI3K-independent even in the absence of serum in astrocytoma cells. SF-268 cells were treated with 50 μM U0126

for 24 h or with wortmanin 100 nM (Wm) for 4 h (with DMSO as carrier). Cells were then subjected to 4 h hypoxia, then the complete medium was removed and the cells incubated in serum-free medium for 3 h (for starvation). Cells were then lysed and cell lysates blotted for VEGF-A or β-actin for loading control."

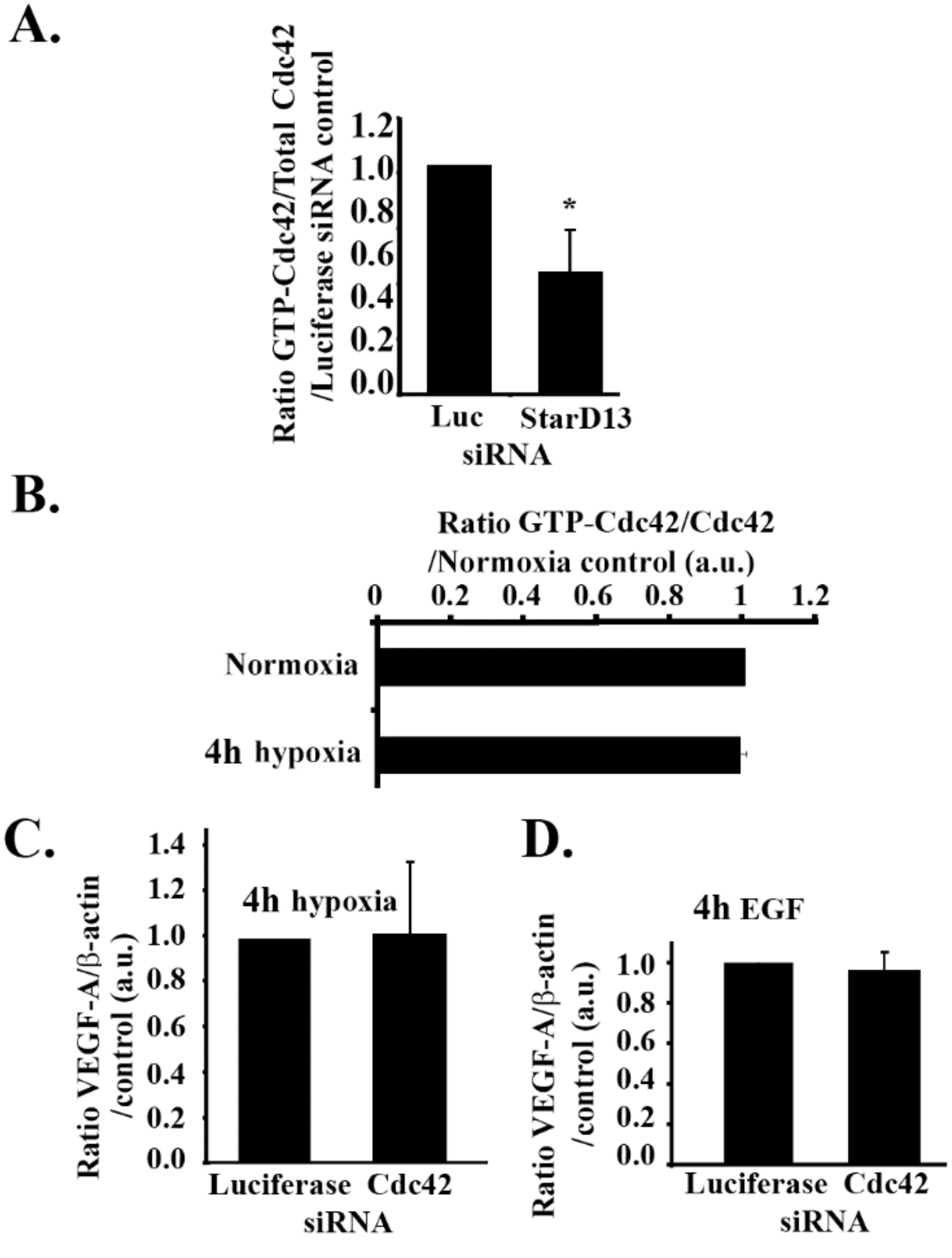

Supplemental Figure S5: Hypoxia does not affect the activation of Cdc42 in astrocytoma cells. **(A)** SF-268 cells were transfected with luciferase or StarD13 siRNA for 72 h then lysed. The lysates were then subjected to a Cdc42 pull-down assay (GTP-bound Cdc42 was pulled down with CRIB). Graph is a quantitation of active Cdc42 normalized to total Cdc42 and expressed as fold change compared to luciferase siRNA control.

(B) SF-268 cells were subjected to hypoxia for 4 h, or left in normoxia and lysed. The lysates were then subjected to a Cdc42 pull-down assay as in (A). Graph is a quantitation of the bands in the upper gel (active Cdc42) normalized to total Cdc42 and expressed as fold change compared to time zero (normoxia). **(C/D)** SF-268 cells were transfected with luciferase siRNA for control of with Cdc42 siRNA for 72 h. Cells were then subjected to 4 h hypoxia or left in normoxia (C) or treated with EGF for 4 h or left untreated, after starvation (D). Cells were then lysed and cell lysates blotted for VEGF-A or β-actin for loading control The graphs are quantitations for the VEGF bands normalized to actin and expressed as fold change compared to control (Luciferase siRNA). The data are the mean ± SEM from 3 different experiments (n=3). * $p < 0.05$ indicates statistically significant differences.
